# Supplementary material for: Structural determinants of microtubule minus end preference in CAMSAP CKK domains
Source: Nat Commun. 2019 Nov 20;10:5236. doi: 10.1038/s41467-019-13247-6 (PMC6868217; doi:10.1038/s41467-019-13247-6)
Supplement: Supplementary file 1 — Supplementary Information [file 41467_2019_13247_MOESM1_ESM.pdf]

Supplementary Information

Structural determinants of microtubule minus end preference in CAMSAP CKK domains

Joseph Atherton et al.

Supplementary Table 1. Cryo-EM reconstruction information, model refinement statistics and model geometry. Data collection and processing information for the HsCKK and NgCKK 13-protofilament and 14-protofilament MT datasets and model refinement statistics for the tubulin + CKK asymmetric unit models built into their respective symmetrized electron density maps.

	HsCKK 13pf (EMDB-4643, PDB: 6QUS)	HsCKK 14pf (EMDB-4654, PDB: 6QVJ)	NgCKK 13pf (EMDB-4644, PDB: 6QUY)	NgCKK 14pf (EMDB-4650, PDB: 6QVE)
**Data collection and processing**				
**Pixel size (Å)**	**1.39Å**	**1.39Å**	**1.39Å**	**1.39Å**
**Symmetry imposed***	**Pseudo-helical**	**Pseudo-helical**	**Pseudo-helical**	**Pseudo-helical**
**Number of micrographs**	**1075**	**1075**	**386**	**386**
**Initial particle images (no.)**	**82,666**	**82,666**	**60,890**	**60,890**
**Final particle images (no.)**	**36,342**	**33,419**	**27,635**	**28,421**
**Symmetrised Map resolution (Å)**	**3.7Å**	**3.8Å**	**3.8Å**	**3.7Å**
**FSC threshold†**	**Independent half-map FSC 0.143**	**Independent half-map FSC 0.143**	**Independent half-map FSC 0.143**	**Independent half-map FSC 0.143**
**Map resolution range (Å)**	**3.6-4.4Å**	**3.7-4.5Å**	**3.7-4.5Å**	**3.6-4.4Å**
**Refinement**				
**Initial models used (PDB)**	**5LZN, 1UGJ**	**5LZN, 1UGJ**	**5LZN, 1UGJ**	**5LZN, 1UGJ**
**Refinement resolution (Å)**	**3.7**	**3.8**	**3.8**	**3.7**
**$FSC_{average}$‡**	**0.82**	**0.82**	**0.86**	**0.87**
**Map local sharpening *B*-factor ($Å^2$)**	**-75**	**-75**	**-70**	**-70**
**Model composition**				
**Nonhydrogen atoms**	**45,127**	**44,830**	**44,829**	**44,645**
**Protein residues**	**5,650**	**5,612**	**5,624**	**5,600**
**Ligands**	**18**	**18**	**18**	**18**
**R.m.s. deviations§**				
**Bond lengths (Å)**	**0.01**	**0.01**	**0.01**	**0.01**
**Bond angles (°)**	**0.89**	**0.98**	**0.90**	**0.89**
**Validation#**				
**MolProbity score**	**1.67**	**1.76**	**1.71**	**1.71**
**Clashscore**	**5.06**	**5.78**	**5.07**	**5.4**
**Poor rotamers (%)**	**0.2%**	**0.4%**	**0.2%**	**0.25%**
**Ramachandran plot#**				
**Favored (%)**	**93.99**	**92.99**	**93.52**	**93.15**
**Allowed (%)**	**6.01**	**7.01**	**6.48**	**6.85**
**Outliers (%)**	**0**	**0**	**0**	**0**

*MTs exhibit pseudo-helical symmetry due to a symmetry break at the seam. 13-fold or 14-fold pseudo-helical symmetry was applied to the 13-protofilament and 14-protofilament MT reconstructions respectively.

†The resolution value at the gold-standard Fourier Shell Correlation (FSC) 0.143 criterion between independently refined half-maps is shown for the central 15% of the reconstructions along the helical axis (isolated with a soft mask).

‡Average Fourier Shell Correlation between the model and the symmetrized electron density map of the asymmetric unit (calculated around model atoms only).

§Root-mean-square (R.m.s) deviations of bond lengths or angles in the model.
#As defined by the MolProbity validation server[1].

Supplementary Table 2. Helical parameters for CKK-MT reconstructions. 3-start rise and twist were calculated directly from the reconstructions in RELION v3.0's helical mode[2-4]. Intradimer, interdimer and dimer repeat distances were calculated directly from the refined models in Chimera[5]. Dimer Skew was measured as azimuthal (ϕ) angle change per dimer in the aligned data sets as described for Figure 4b.

	**3-start rise (Å)**	**3-start twist (°)**	**Intradimer distance (Å)**	**Interdimer distance (Å)**	**Dimer distance (Å)**	**Dimer Skew (°)**
**CAMSAP1-CKK 13pf-taxol**	9.49	-27.65	41.3	41.00	82.3	0.50
**N.Grub-CKK 13pf-taxol**	9.47	-27.67	41.1	40.8	82.0	0.14
**CAMSAP1-CKK 14pf-taxol**	8.80	-25.72	41.2	40.8	82.0	-0.04
**N.Grub-CKK 14pf-taxol**	8.80	-25.72	41.1	40.9	82.0	-0.28

Supplementary Table 3. Primer sequences used for chimeric and truncated CKK constructs.

**Primer name**	**Primer sequence**
NgCKK Delta N Fw	GTACAAGTCCGGACTCAGATCTAAGACGAACAAACTGCTGATAAAGAATGCTCT
NgCKK Delta N Rv	GGGCCCGCGGTACCGCTACCTCTTTAT
NgCKK Delta C Fw	GTACAAGTCCGGACTCAGATCTATTCAACAGAAGC
NgCKK Delta C Rv	GGGCCCGCGGTACCGCTactTAATGGCAGC
NgCKK Swap N 1Fw	GTACAAGTCCGGACTCAGATCTGCATCTTCCCTGGCCTCAG
NgCKK Swap N 1Rv	GTTCGTCTTACTACTGGGCTCCTTAAAGAGCTTGG
NgCKK Swap N 2Fw	CCCAGTAGTAAGACGAACAAACTGCTGATAAAGAATGCTCT
NgCKK Swap C Rv	GGGCCCGCGGTACCGTCATTTACGAGTCTGGGCCTTCTTTGGCACTGCAGGCCGCTTGGGCTTAATGGCAGC

SUPPLEMENTARY FIGURES

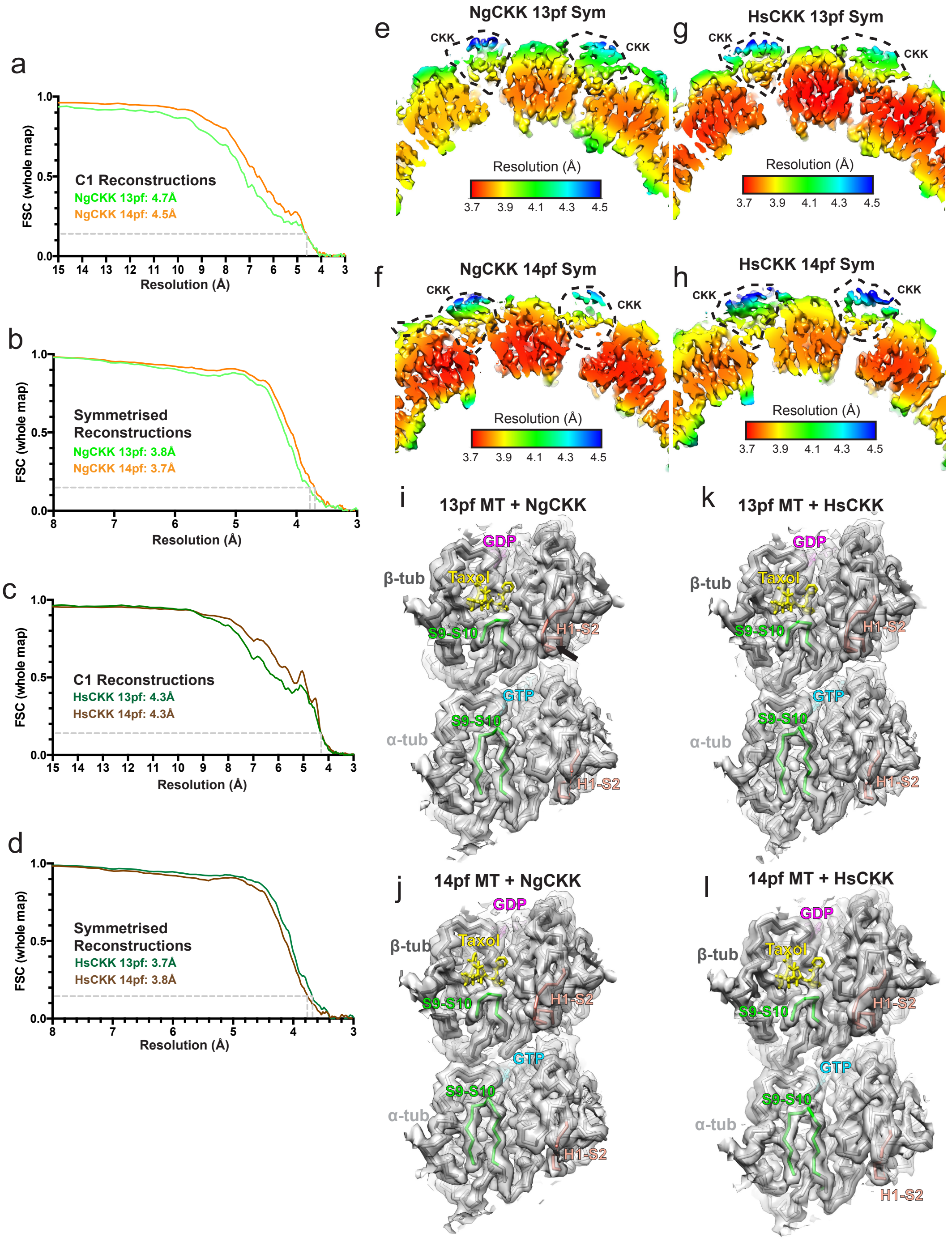

Supplementary Figure. 1. Evaluation of the resolutions of the CKK-MT reconstructions. a) FSC curves for the NgCKK-MT 13- (green) and 14-protofilament (orange) C1 reconstructions, with the 0.143 cut-off indicated; b) FSC curves for the NgCKK-MT 13- (green) and 14-protofilament (orange) symmetrised reconstructions, with the 0.143 cut-off indicated; c) FSC curves for the HsCKK-MT 13- (green) and 14-protofilament (brown) C1 reconstructions, with the 0.143 cut-off indicated; d) FSC curves for the HsCKK-MT 13- (green) and 14-protofilament (brown) symmetrised reconstructions, with the 0.143 cut-off indicated; e) Local resolution depiction using RELION v3.0’s local resolution determination

of a slice through the NgCKK-MT symmetrised 13-protofilament reconstruction; f) Local resolution depiction of the NgCKK-MT symmetrised 14-protofilament reconstruction; g) Local resolution depiction of the HsCKK-MT symmetrised 13-protofilament reconstruction; h) Local resolution depiction of the HsCKK-MT symmetrised 14-protofilament reconstruction viewed from the MT minus end; i) Lumenal face of the tubulin dimer of the asymmetric unit of the NgCKK-MT symmetrised 13-protofilament reconstruction; j) Lumenal face of the tubulin dimer of the NgCKK-MT symmetrised 14-protofilament reconstruction; k) Lumenal face of the tubulin dimer of the HsCKK-MT symmetrised 13-protofilament reconstruction; l) Lumenal face of the tubulin dimer of the HsCKK-MT symmetrised 14-protofilament reconstruction; i-l) all reconstructions show well defined density for the H1-S2 and S9-S10 loops (coloured pink and green respectively), which are distinct in α- and β-tubulin.

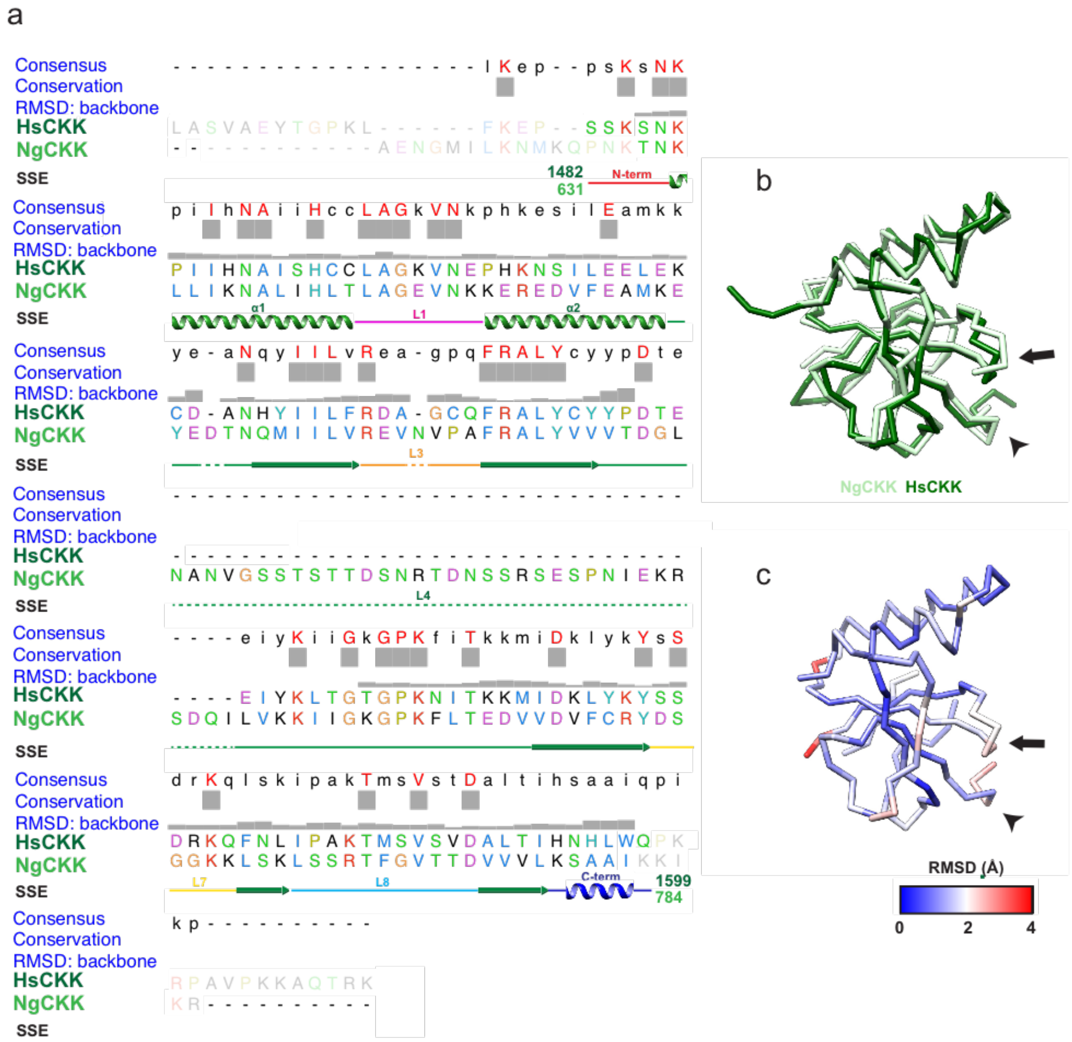

Supplementary Figure 2. Comparison of NgCKK and HsCKK primary and 3D structures. a) Sequence alignment of HsCKK and NgCKK (extracted from our previous larger CKK domain sequence alignment[6]) annotated in UCSF Chimera[5] with sequence consensus, conservation, backbone RMSD and secondary structure elements. The N- and C-terminal residue number for the CKK domain within each full-length protein are also indicated; b) Overlay of aligned NgCKK (light green) and HsCKK (dark green) backbone

models viewed from the MT lumen; c) Backbone RMSD between NgCKK and HsCKK shown on the NgCKK structure. In both b) and c), the arrow indicates loop3 and the arrowhead indicates the CKK C-terminus, regions of notable structural divergence between NgCKK and HsCKK.

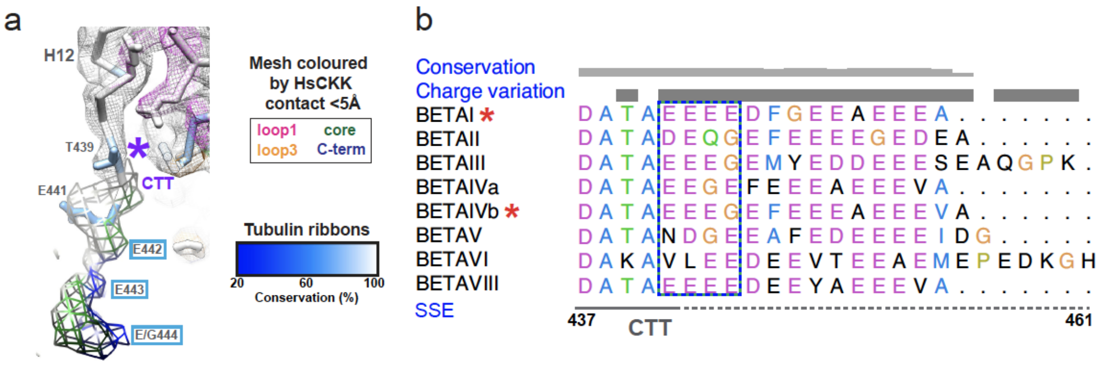

Supplementary Figure 3. Visualisation of β-tubulin's CTT interaction with HsCKK is likely facilitated by the homogeneity of tsA201 cell tubulin. a) Density isolated from the HsCKK-MT cryo-EM reconstruction, with the region corresponding to the ordered β-tubulin C-terminal tail (CTT) shown as thick mesh; the sequence of the well-ordered CTT region in tsA201-cell tubulin is shown with unmodelled residues labelled in light blue boxes; the mesh is coloured according to the closest secondary structural element of HsCKK (see key); b) Human β-tubulin isoform sequences (letters coloured in Clustal-X style by residue subtype) for C-terminal tail residues 438-444, showing that the BETAI and BETAIVb isoforms present in tsA201 cell tubulin (indicated by a red *) only differ in their final residue. Blue boxed region indicates residues that were not fully modelled, although density corresponding to this region is visualized and forms contacts with both the CKK core and C-terminus.

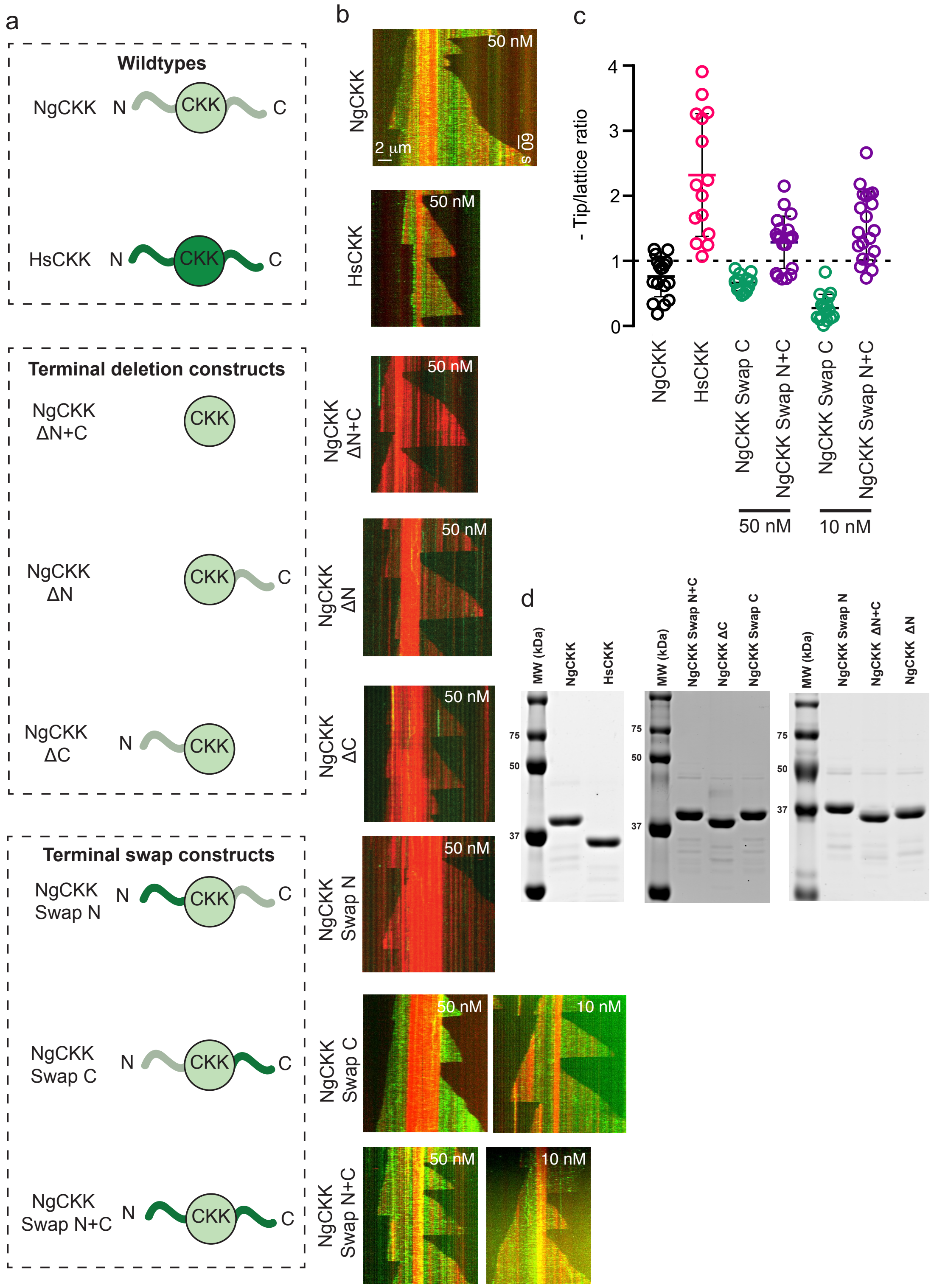

Supplementary Figure 4. The absence of minus-end recognition by NgCKK is not a gain-of-function mediated by its termini. a) Schematic of CKK domain truncations and chimeras used; b) Representative kymographs illustrating the binding behaviour of indicated proteins with microtubules; GFP-tagged CKK proteins were added at 50nM unless otherwise indicated, scale bars: horizontal 2 μm, vertical 60 s; c) Quantification of microtubule minus end to lattice ratio for the GFP-CKK constructs where this could be measured; n = 17, 15, 16

and 20 MTs for 50 nM NgCKK, HsCKK, Swap C and Swap N+C, n = 16 and 20 for 10nM Swap C and Swap N+C respectively; N=2 independent experiments; all data points are plotted and bars represent mean ± SD; d) SDS-PAGE Coomassie-stained gels showing the purified proteins used in these assays; source data are provided as a Source Data file.

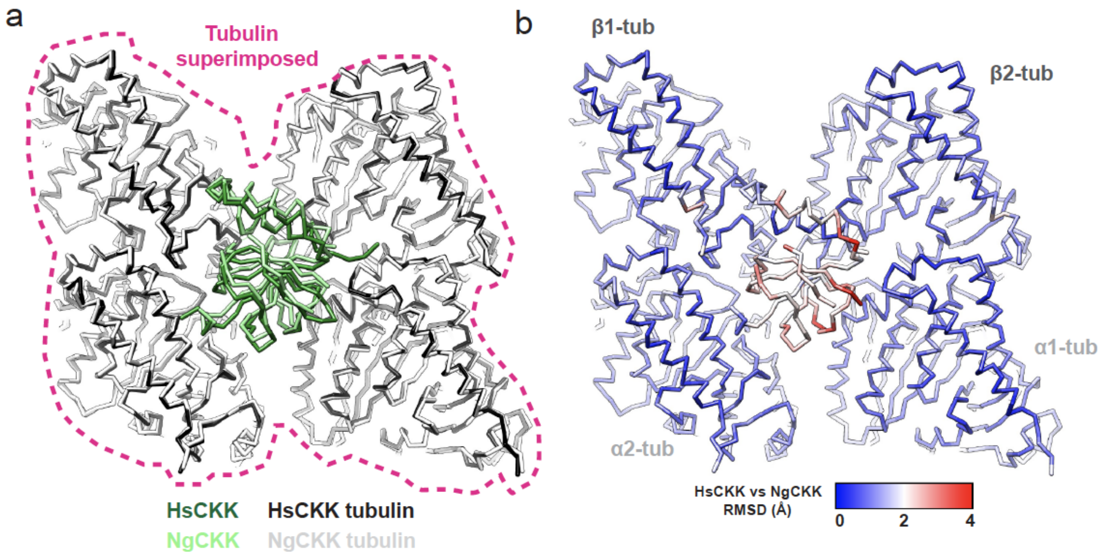

Supplementary Figure 5. Structural differences in MT interaction between NgCKK and HsCKK. a) Backbone models of tubulin dimer pairs from the NgCKK-MT (light grey) and HsCKK-MT (dark grey) structures align very well, while the CKK domains (NgCKK, light green; HsCKK, dark green) exhibit differences especially adjacent to the α-tubulins (annotated in detail in Fig. 2a); b) Backbone RMSD between these models shown on the NgCKK-MT model; models are viewed from the outside MT surface in a) and b).

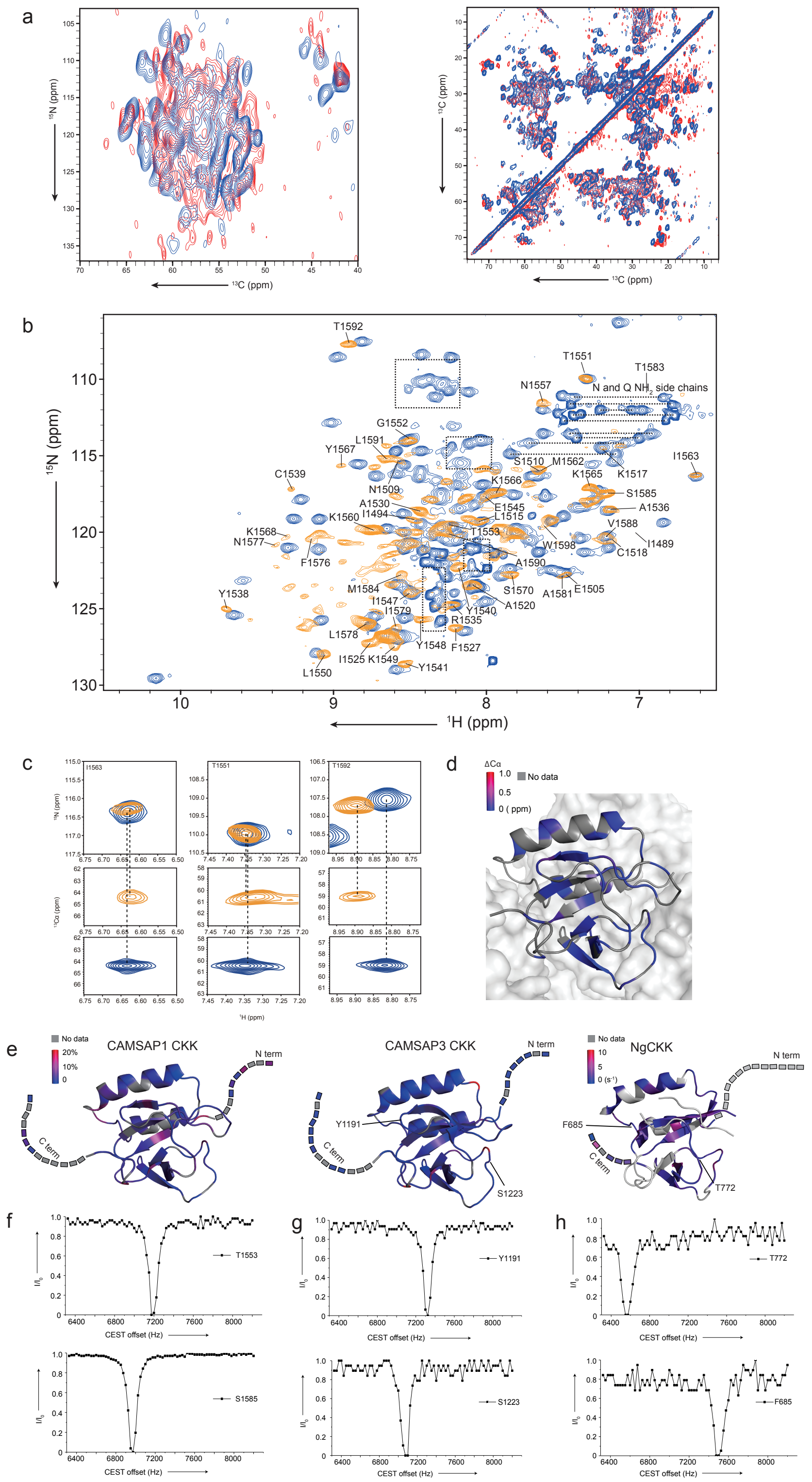

Supplementary Figure 6. Rigidity of the HsCKK domain revealed by high-resolution NMR. a) Comparison of solid-state NMR spectra obtained on WT (red, CAMSAP3 CKK) and mutant (blue, CAMSAP1 CKK N1492A) CKK. Data are shown from 2D NCA (left panel) and 2D PDSD spectra (right panel); b) NMR Chemical-shift perturbations of CAMSAP1 CKK N1492A upon binding to MTs. 2D NH correlations are shown for free CAMSAP1 CKK N1492A in solution (blue) and MT bound CKK (orange). For clarification,

NH_2 correlations from the side chains of N and Q residues are indicated by dashed lines. Furthermore, signals from purification tags are highlighted (boxes) that do not appear in the ssNMR NH correlation spectrum (orange) of the MT-bound CKK. The 48 transferred assignments based on solution-state NMR data (where 124 residues in total were assigned) are shown on the spectrum. Note that the signals for I1489 and T1583 are not visible at the chosen contour level but appear at lower contour levels in the NH spectrum; c) Spectral cutouts comparing solid-state NMR signal sets of free (blue) and MT bound (orange) HsCKK_N1492A for three selected residues. Top panels show the zoom-in regions from the 2D ^{15}N-^{1}H correlated spectra, while the middle and bottom panels reflect regions from 3D CANH (solid state) and HNCA (liquid state) spectra at corresponding ^{15}N chemical shifts, respectively. See panel b for full spectra. d) Chemical-shift perturbations of Cα atoms, coloured by difference in ppm, arising from HsCKK_N1492A binding to MTs mapped on the structure of CKK-MT complex (PDB: 5M5C); the CKK domain is depicted in a ribbon representation while the MT surface is shown as a space-filling model; e) Protein Dynamic-sensitive NMR profiles for wildtype CKK domains. Comparison of CAMSAP1 CKK (left panel, taken from PDB: 5M54), CAMSAP3 CKK (middle panel, taken from PDB: 5M50) and NgCKK (right panel, taken from PDB 6QUS) transverse relaxation rate changes with same CPMG setting as Fig. 5a; f) Solution CEST profiles of free HsCKK_N1492A shown for residues T1553 and S1585, which showed significant chemical shift perturbation upon binding to MTs (see Fig. 5a); g) CEST profiles of Y1191 and S1223 from CAMSAP3 CKK exhibiting similar dynamics profiles as shown in panel e; h) CEST profiles of T772 and F685 from NgCKK exhibiting similar dynamics profiles as shown in panel f. Source data are provided as a Source Data file.

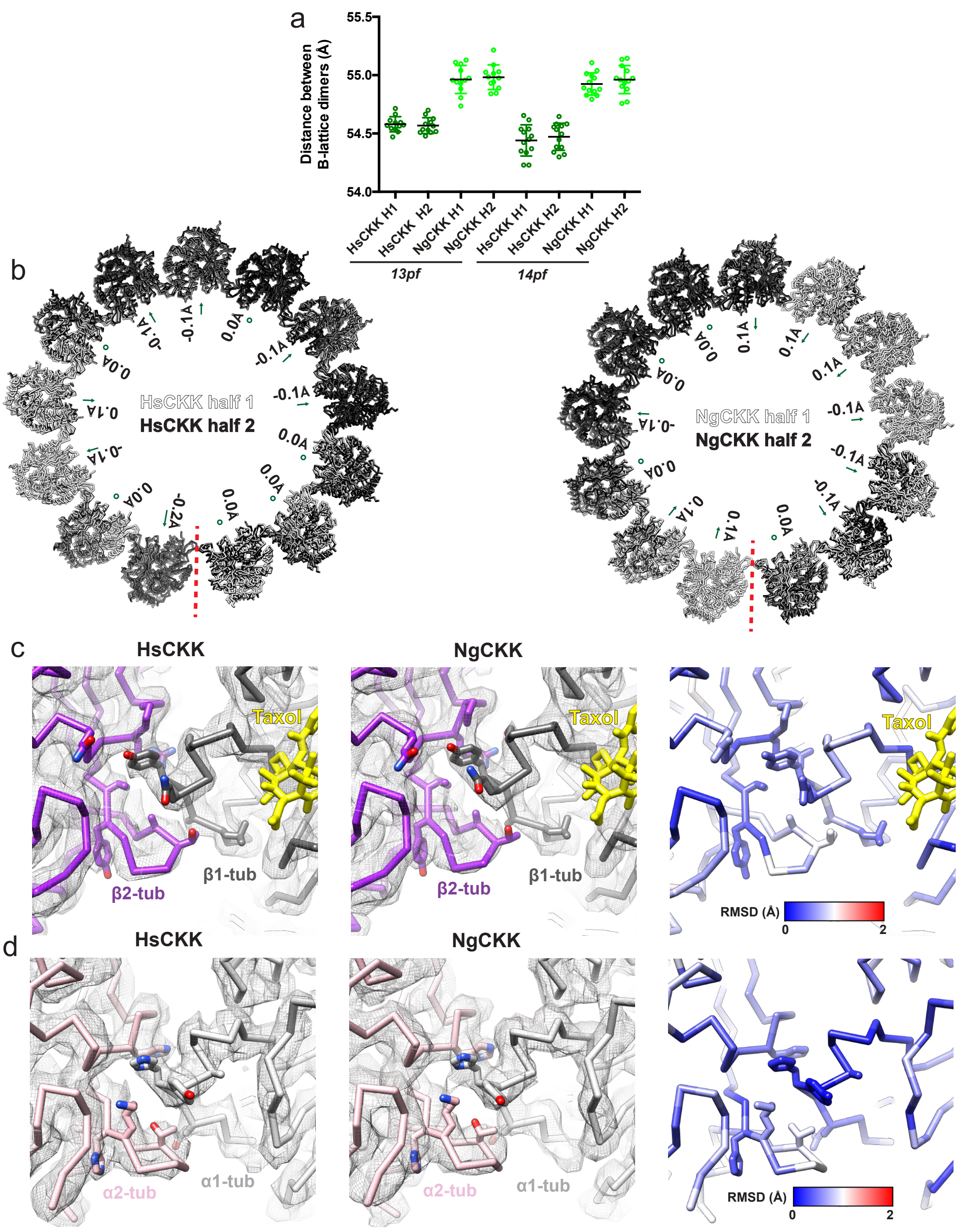

Supplementary Figure 7. HsCKK MT remodelling does not cause detectable differences in the inter-protofilament lateral contacts.

a) The distance between the centre of masses of each pair of adjacent B-lattice dimers was measured from models fitted into the C1 independent half-maps from 13- and 14-protofilament HsCKK and NgCKK data; all data points are plotted and bars represent mean ± SD; there are no significant differences between half-maps within each dataset ($p>0.05$, t-tests); source data are provided as a Source Data file; b) a single turn of models docked within aligned C1 independent half-maps from either 13-protofilament left, HsCKK, or right, NgCKK datasets are shown viewed from the minus end; arrows indicate the irregular shift of individual protofilaments; there are no differences between half-maps within each dataset; c)

Comparison of the β-tubulin lateral contacts viewed from the MT lumen in the 13-protofilament, left, HsCKK reconstruction and model, middle, NgCKK reconstruction and model, right, backbone RMSD of this region, showing that the remodelling of the MT architecture does not cause detectable difference in the β-tubulin interprotofilament lateral contacts; β1-tubulin is coloured dark grey, β2-tubulin is coloured purple and taxol is shown in yellow; d) Comparison of the α-tubulin lateral contacts viewed from the MT lumen in the 13-protofilament, left, HsCKK reconstruction and model, middle, NgCKK reconstruction and model, right, backbone RMSD of this region, showing that the remodelling of the MT architecture does not cause detectable difference in the α-tubulin interprotofilament lateral contacts; α1-tubulin is coloured light grey and α2-tubulin is coloured pink.

References

1. Chen, V.B. *et al*. MolProbity: all-atom structure validation for macromolecular crystallography. *Acta Crystallogr D Biol Crystallogr* **66**, 12-21 (2010).
2. He, S. & Scheres, S.H.W. Helical reconstruction in RELION. *J Struct Biol* **198**, 163-176 (2017).
3. Scheres, S.H. RELION: implementation of a Bayesian approach to cryo-EM structure determination. *J Struct Biol* **180**, 519-530 (2012).
4. Zivanov, J. *et al*. New tools for automated high-resolution cryo-EM structure determination in RELION-3. *Elife* **7** (2018).
5. Pettersen, E.F. *et al*. UCSF Chimera--a visualization system for exploratory research and analysis. *J Comput Chem* **25**, 1605-1612 (2004).
6. Atherton, J. *et al*. A structural model for microtubule minus-end recognition and protection by CAMSAP proteins. *Nat Struct Mol Biol* **24**, 931-943 (2017).
