# Supplementary material for: Structural determinants of microtubule minus end preference in CAMSAP CKK domains
Source: Nat Commun. 2019 Nov 20;10:5236. doi: 10.1038/s41467-019-13247-6 (PMC6868217; doi:10.1038/s41467-019-13247-6)
Supplement: Supplementary file 2 — Description of Additional Supplementary Files [file 41467_2019_13247_MOESM2_ESM.pdf]

Description of Additional Supplementary Files

File name: Supplementary Movie 1

Description: Comparison of NgCKK and HsCKK binding positions on two laterally adjacent tubulin dimers. The pair of neighbouring tubulin dimers in 13-protofilament HsCKK and NgCKK models were superimposed and a morph created between the two, demonstrating structural and positional differences between the HsCKK and NgCKK relative to the MT lattice. The CKK models are coloured according to SSE/loop colour scheme in Fig 1.

File name: Supplementary Movie 2

Description: NgCKK is minimally displaced from its binding site in 14- compared to 13-protofilament MTs. A transverse view from the MT plus end through 3 protofilaments from the NgCKK-MT 13- morphing to 14- protofilament models (backbone representation) superimposed on the central protofilament. This shows how the adjacent protofilaments adopt a shallower relative angle in 14-protofilament MTs and reveals the minimal displacement of bound NgCKK in response to this change in lateral protofilament curvature. NgCKK is shown in light green, β-tubulin in dark grey, α-tubulin in light grey.

File name: Supplementary Movie 3

Description: HsCKK is displaced from its binding site in 14- compared to 13-protofilament MTs, demonstrating its greater sensitivity to microtubule lateral curvature. A transverse view from the MT plus end through 3 protofilaments from the HsCKK-MT 13- morphing to 14- protofilament models (backbone representation) superimposed on the central protofilament. This shows how the adjacent protofilaments adopt a shallower relative angle in 14-protofilament MTs and reveals the displacement of bound HsCKK in response to this change in lateral protofilament curvature. HsCKK is shown in green, β-tubulin in dark grey, α-tubulin in light grey.
