# Supplementary material for: Structural determinants of microtubule minus end preference in CAMSAP CKK domains
Source: Nat Commun. 2019 Nov 20;10:5236. doi: 10.1038/s41467-019-13247-6 (PMC6868217; doi:10.1038/s41467-019-13247-6)
Supplement: Supplementary file 6 — Reporting Summary [file 41467_2019_13247_MOESM6_ESM.pdf]

nature research

Corresponding author(s): Joseph Atherton; Marc Baldus; Carolyn A. Moores

Last updated by author(s): Mar 22, 2019

Reporting Summary

Nature Research wishes to improve the reproducibility of the work that we publish. This form provides structure for consistency and transparency in reporting. For further information on Nature Research policies, see Authors & Referees and the Editorial Policy Checklist .

Statistics

For all statistical analyses, confirm that the following items are present in the figure legend, table legend, main text, or Methods section.

n/a	Confirmed	
	x	The exact sample size (*n*) for each experimental group/condition, given as a discrete number and unit of measurement
x		A statement on whether measurements were taken from distinct samples or whether the same sample was measured repeatedly
	x	The statistical test(s) used AND whether they are one- or two-sided<br>*Only common tests should be described solely by name; describe more complex techniques in the Methods section.*
x		A description of all covariates tested
x		A description of any assumptions or corrections, such as tests of normality and adjustment for multiple comparisons
	x	A full description of the statistical parameters including central tendency (e.g. means) or other basic estimates (e.g. regression coefficient) AND variation (e.g. standard deviation) or associated estimates of uncertainty (e.g. confidence intervals)
x		For null hypothesis testing, the test statistic (e.g. *F*, *t*, *r*) with confidence intervals, effect sizes, degrees of freedom and *P* value noted<br>*Give P values as exact values whenever suitable.*
x		For Bayesian analysis, information on the choice of priors and Markov chain Monte Carlo settings
x		For hierarchical and complex designs, identification of the appropriate level for tests and full reporting of outcomes
x		Estimates of effect sizes (e.g. Cohen's *d*, Pearson's *r*), indicating how they were calculated

Our web collection on statistics for biologists contains articles on many of the points above.

Software and code

Policy information about availability of computer code

Data collection	TIRF microscopy was performed on an inverted research microscope equipped with a EMCCD camera, and controlled with MetaMorph 7.7 software (Molecular Devices). Manual cryo-EM data collection was performed using Serial-EM. NMR experiments were performed on a 600 MHz spectroscopy, a 950 MHz standard-bore spectrometer and an 800 MHz wide-bore spectrometer (Bruker Biospin), and controlled with TopSpin v3.2 software (Bruker Biospin).
Data analysis	Movie frames were aligned using Motioncorr2; cryo-EM data were processed using RELION v3.0; models were built using Coot and refined in real-space using Phenix; NMR data were analyzed using NMRFAM-SPARKY 1.4.

For manuscripts utilizing custom algorithms or software that are central to the research but not yet described in published literature, software must be made available to editors/reviewers. We strongly encourage code deposition in a community repository (e.g. GitHub). See the Nature Research guidelines for submitting code & software for further information.

Data

Policy information about availability of data

All manuscripts must include a data availability statement. This statement should provide the following information, where applicable:

- Accession codes, unique identifiers, or web links for publicly available datasets
- A list of figures that have associated raw data
- A description of any restrictions on data availability

The 13-protofilament and 14-protofilament HsCKK and NgCKK-MT models along with their corresponding electron density maps are deposited in the PDB. The PDB codes are as follows: 13-protofilament HsCKK-MT, PDB: 6QUS, 14-protofilament HsCKK-MT, PDB:6QVJ, 13-protofilament NgCKK-MT, PDB: 6QUY, 14-protofilament NgCKK-MT, PDB:6QVE. The EMDB codes (C1 reconstruction and symmetrised asymmetric unit) are as follows: 13-protofilament HsCKK-MT, EMDB-4643, 14-protofilament HsCKK-MT, EMDB-4654, 13-protofilament NgCKK-MT, EMDB-4644,14-protofilament NgCKK-MT, EMDB-4650. All supporting data are available from the authors on request, and/or are available in the manuscript itself.

Field-specific reporting

Please select the one below that is the best fit for your research. If you are not sure, read the appropriate sections before making your selection.

- [x] Life sciences
- [ ] Behavioural & social sciences
- [ ] Ecological, evolutionary & environmental sciences

For a reference copy of the document with all sections, see nature.com/documents/nr-reporting-summary-flat.pdf

Life sciences study design

All studies must disclose on these points even when the disclosure is negative.

Sample size	No calculation to pre-determine sample size was performed; sample size is linked to the resolution of the cryo-EM reconstructions as presented in Supplementary Table 1.
Data exclusions	No data were excluded
Replication	Not relevant to study.
Randomization	Not relevant to study.
Blinding	Not relevant to study.

Reporting for specific materials, systems and methods

We require information from authors about some types of materials, experimental systems and methods used in many studies. Here, indicate whether each material, system or method listed is relevant to your study. If you are not sure if a list item applies to your research, read the appropriate section before selecting a response.

Materials & experimental systems

n/a	Involved in the study	
x		Antibodies
x		Eukaryotic cell lines
x		Palaeontology
x		Animals and other organisms
x		Human research participants
x		Clinical data

Methods

n/a	Involved in the study	
x		ChIP-seq
x		Flow cytometry
x		MRI-based neuroimaging
